# Large-scale DNA Barcode Library Generation for Biomolecule Identification in High-throughput Screens

**DOI:** 10.1038/s41598-017-12825-2

**Published:** 2017-10-24

**Authors:** Eli Lyons, Paul Sheridan, Georg Tremmel, Satoru Miyano, Sumio Sugano

**Affiliations:** 1 Laboratory of Functional Genomics, Department of Computational Biology and Medical Science, School of Frontier Sciences, University of Tokyo, Tokyo, 108-8639 Japan; 2 Department of Active Life Promotion Science, Graduate School of Medicine, Hirosaki University, Hirosaki, 036-8562 Japan; 3 Laboratory of DNA Information Analysis, Human Genome Center, Institute of Medical Science, University of Tokyo, Tokyo, 108-8639 Japan

## Abstract

High-throughput screens allow for the identification of specific biomolecules with characteristics of interest. In barcoded screens, DNA barcodes are linked to target biomolecules in a manner allowing for the target molecules making up a library to be identified by sequencing the DNA barcodes using Next Generation Sequencing. To be useful in experimental settings, the DNA barcodes in a library must satisfy certain constraints related to GC content, homopolymer length, Hamming distance, and blacklisted subsequences. Here we report a novel framework to quickly generate large-scale libraries of DNA barcodes for use in high-throughput screens. We show that our framework dramatically reduces the computation time required to generate large-scale DNA barcode libraries, compared with a naїve approach to DNA barcode library generation. As a proof of concept, we demonstrate that our framework is able to generate a library consisting of one million DNA barcodes for use in a fragment antibody phage display screening experiment. We also report generating a general purpose one billion DNA barcode library, the largest such library yet reported in literature. Our results demonstrate the value of our novel large-scale DNA barcode library generation framework for use in high-throughput screening applications.

## Introduction

A DNA barcode, or barcode for short, is a DNA sequence commonly used to identify a target molecule during DNA sequencing, but may also be used for other purposes such as the disruption of gene function or encoding information into a larger DNA region. In the past decade, libraries of DNA barcodes have found use in chemical compound screens^1,2^, the study of clonal diversity^3^, and genomic screens^4,5^. DNA barcoding technology has shown utility in applications such as the discovery of new drug candidates^2^ and elucidating protein interactions in yeast^6^.

DNA barcode libraries can be categorized into two groups, randomly generated DNA barcode libraries that are generated by physically assembling oligos in pools, and rationally designed DNA barcode libraries that are design in silico and then manufactured. Randomly generated DNA libraries may be used for practical reasons such as cost, however, rationally designing DNA barcode libraries can have the advantage of being more robust against misidentification due to DNA sequencing and synthesis errors^7^. Technological constraints have limited the size of the DNA barcode libraries used in screening experiments with the largest sized rationally designed DNA barcode library to date consisting of 240,000 barcodes^8^. But continuing trends in DNA reading and writing technologies are opening up new applications for large-scale DNA barcode libraries. The first trend is an increase in the number of short reads made available by Next Generation Sequencing (NGS) technology^9^. The second is the decreasing cost of manufacturing synthetic DNA^10^. Together these trends make it possible to perform high-throughput experiments using synthetic DNA libraries in combination with screening experiments and NGS technology. Kosuri *et al*.^10^ conjecture that the cost of gene synthesis could become on par with oligo pools (1 USD per 10^3^ to 10^5^ bp), which would allow for DNA barcodes to be used in practical applications in which a designed DNA sequence and unique DNA barcode are designed and manufactured together in synthetic DNA. In this case it would be beneficial for the DNA barcode sequence and biomolecule sequence it identifies to be known in advance for identification purposes, and for the barcodes to be designed in a robust manner. Applications include the construction and screening of synthetic large-scale barcoded fragment antibody libraries and barcoded synthetic genomes. Therefore we expect methods for the fast and robust design of large-scale barcode libraries (i.e. libraries consisting of more than 10^6^ barcodes) will be needed for biologists to apply synthetic DNA and DNA sequencing technologies to their research.

A number of methods for generating customizable DNA barcode libraries have been proposed. Barcode Generator^11^ and nxCode^12^ are free tools that have been employed by their creators to generate publicly available libraries consisting of up to 96 and 587 barcodes, respectively. More sophisticated barcode library design methods based on error-correcting codes have been proposed for correcting substitutions and indel errors inherent to NGS sequencing technologies^7,13–15^. The DNABarcodes R package^16^ implements the error-correcting codes based generation method of Buschmann and Bystrykh^7^. The libraries described in the package manual contain from tens to hundreds of barcodes. The authors of the TagGD software package report generating libraries consisting of 100,000 barcodes in 1.5 to 7 hours, depending on the conditions of customization^17^. More recently Waang *et al*.^18^ introduced a method to generate barcode libraries using particle swarm optimization. Lastly, Xu *et al*.^8^ describe an elaborate barcode library design methodology that they used to generate a library consisting of 240,000 barcodes. While each method is appropriate to the generation of barcode libraries for applications their creators had in mind, none of them were designed to quickly generate the sorts of large-scale barcode libraries required for the high-throughput screening applications we envision.

Here we present a novel framework for the rapid generation of large-scale, customized DNA barcode libraries. The framework we propose can be used to generate large-scale barcode libraries that satisfy a minimum pairwise Hamming distance between barcodes, a maximum homopolymer length, lower and upper GC content limits, and are free from blacklisted sequences. The primary reason why our framework can produce large-scale libraries is that the underlying algorithm combinatorially assembles the individual barcodes in a library from pairs of shorter DNA sequences. This has the effect of increasing the rate of barcode generation by reducing computationally expensive Hamming distance comparisons. A secondary novelty behind the speed of our framework is that the shorter DNA sequences are generated according to a Markov chain model with basic hyper-parameter optimization. In a typical experiment, each barcode in a library functions as a unique identifier, or “target code” for identifying the molecule to which it is paired. But the structure of barcodes generated by our framework has a additional interpretation in the context of pooled experiments. Consider, for example, a binding competition experiment where chemical library A and chemical library B are screened both separately and together for binding affinity against a target antigen. In this case the biologist can take advantage of the barcode substructure that has a batch code and target code. Using our barcode framework all the chemicals in library A would be tagged with barcodes that all have the same batch code in their substructure specific to library A, with each barcode in library A containing a unique target code in their substructure. All the chemicals in library B would be tagged with barcodes that contain a different batch code in their substructure from the barcodes used with library A, but the target codes used in library A can be reused in library B. This has an additional advantage for the working biologist that already has the physical DNA of barcodes that they would like to mix with an additional batch of barcodes for a new experiment. In this case a biologist using our framework would only need to generate one additional batch code that passes the filters and would not have to generate additional target codes.

This paper is structured in the following way. In the Methods section we describe our framework that combines DNA barcode structure design, a Markov chain model for generating nucleotide sequences, and hyper-parameter optimization by grid search. In the Results section we test our framework and show it outperforms naïve methods that use a conventional barcode structure and random nucleotide generation when generating a large-scale barcode libraries consisting of one million barcodes for use in a fragment antibody phage display screening experiment. Finally, in the Conclusion section we comment on future research directions and practical matters for applications of DNA barcodes in research.

## Methods

In this section we describe our DNA barcode library generation framework. The method is used to generate a DNA barcode library consisting of *N* barcodes of length *L* bp subject to the following configurable constraints (filters):

**Hamming filter:** The minimum global Hamming distance permitted between barcode pairs. This is denoted by *d*.

**Homopolymer filter:** The maximum homopolymer length allowable in a barcode. This is denoted by *m*.

**GC-content filter:** The lower and upper limits on the GC-content of a barcode.

**Blacklist filter:** A “blacklist” of proscribed DNA sequences.

When we speak of a barcode library in this paper we mean a collection of *N* barcodes of length *L* bp generated within this framework.

### DNA Barcode Structure

**Figure 1:**
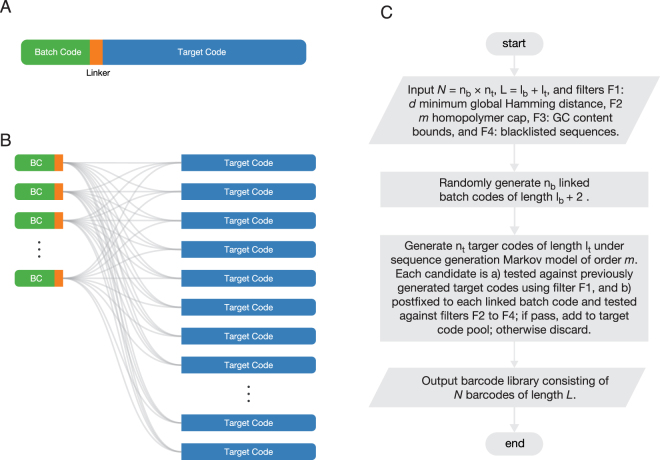
Barcode structure and library generation overview. (**A**) An individual barcode consists of a batch code, a linker, and a target code. Batch codes serve as labels for different experiments and are appended with linkers for technical reasons that will be made apparent in the main text. Target codes serve as labels for particular targets in a given batch. (**B**) A barcode library is constructed by appending linked batch codes with target codes in all possible combinations. (**C**) Flowchart of the main steps involved in the generation of a barcode library within our framework. See main text for details.

The generic structure of a barcode generated according to our framework is shown in Fig. 1A. There are three components: a *batch code* of length ${\ell }_{b}$ bp, a *linker* of length 2 bp, and a *target code* of length ${\ell }_{t}$. We use the term *linked batch code* to refer collectively to a batch code that is appended with a linker. Thus barcode length may be decomposed as $L={\ell }_{b}+{\ell }_{t}+2$. Barcode structure interpretation differs depending on the experimental context. In the use case when all *N* barcodes in a library are intended for use in a single experiment, each barcode serves to identify a unique target, so that the batch/target interpretation ceases to apply in any meaningful sense. In other words, each barcode functions as a “target code” in its own right. However, in a second use case the batch/target code construct becomes important in the context of pooled experiments where the batch code is used to identify the group to which the target molecule belongs. In this case the batch code functions as a unique identifier of an experiment batch, while a target code serves to identify a unique target within a particular batch. Examples of such applications include competition experiments and Barcode Fusion Genetics^6^. The linker is necessitated by technical considerations that will be made apparent below.

### DNA Barcode Library Generation Overview

The structure of a barcode library is shown in Fig. 1B. In general, a library is composed of *n*_*b*_ linked batch codes and *n*_*t*_ target codes. The *N* = *n*_*b*_ × *n*_*t*_ barcodes in the library are made up from all pairwise concatenations of the linked batch codes with the target codes. Therefore, in a pooled experimental setting, *n*_*b*_ corresponds to the maximum number of different experiments, and *n*_*t*_ the maximum number of targets in a particular experiment.

The workflow of our DNA barcode library generation framework is outlined in Fig. 1C. The algorithm requires input parameters *N* (number of barcodes), *n*_*b*_ (number of batch codes), *n*_*t*_ (number of target codes), *L* (barcode length), ${\ell }_{b}$ (batch code length), ${\ell }_{t}$ (target code length), and generation constraints *d* (minimum Hamming distance), *m* (maximum homopolymer length), GC-content min/max bounds, and a blacklist of proscribed sequences.

Barcode library generation may be broken down into two phases: first, a set of *n*_*b*_ batch codes is produced, and second, a set of *n*_*t*_ target codes are generated in such a manner that each target code when postfixed to any linked batch code passes all the filters. It is this last step of ensuring that all the filters are passed that makes for the computationally intensive part of the generation process. Before we come to that, however, we first turn to the underlying matter of nucleotide sequence generation.

### Nucleotide Sequence Generation

Here we describe a Markov chain model of order *m* for nucleotide sequence generation. The model is used to stochastically generate nucleotide sequences of prescribed length with maximum homopolymer length *m*. This model is important to our DNA barcode generation framework. In practice, the use of this model for nucleotide sequence generation increases the number of candidate barcodes that pass the homopolymer filter, compared to what happens when sequences are generated at random.

Let us formally define the model. Let *S* = {*A*, *C*, *G*, *T*} be the set of nucleotide bases. A nucleotide sequence of length *n* is defined as *x*_1_^*n*^ = *x*_1_ *x*_2_, …, *x*_*n*_ for *n* realisations of *x*_*k*_ ∈ *S* for 1 ≤ *k* ≤ *n* where *x*_*k*_ *x*_*k*+1_ denotes concatenation. Our Markov chain model of order *m* is defined by the transition rule

1 $$\Pr ({x}_{k+1}=i|{H}_{k,m}=h)=(\begin{array}{cc}{\delta }^{h} & \,if\,\,{x}_{k}=i\\ \frac{1-{\delta }^{h}}{3} & \,if\,\,{x}_{k}\ne i\end{array}$$

for a nucleotide *i* ∈ *S* with *H*_*k*,*m*_ = *h* defined as the length of the homopolymer beginning from state *k* − 1 running to at most state *h* − *m* + 1. In the text when we say the *m-homopolymer length at state k* we mean the value of *H*_*k*,*m*_. Random nucleotide sequence generation is achieved by setting *δ* = 1/4 and *m* = 1. It is interesting to note that the *m*-homopolymer length at state *k* is given by

2 $${H}_{k,m}=1+\delta ({x}_{k-1},{x}_{k-2})+\prod _{i,j\in \mathrm{[3]|}i < j}\delta ({x}_{k-i},{x}_{k-j})+\cdots +\prod _{i,j\in [m]|i < j}\delta ({x}_{k-i},{x}_{k-j})$$

where [*a*] = 1, 2, …, *a* and *δ*(*x*_*i*_, *x*_*j*_) is the Kronecker delta of the states *x*_*i*_ and *x*_*j*_. It will also be noted that technically *m* is taken as *min*(*m*, *k*) in practice to account for the start of sequence generation. Finally we also implemented a model with *δ* ^*α*^ with *α* > 0 at great cost of our time and energy, but it did not turn out to perform significantly better than our simpler model.

### Batch Code Generation

Here we describe the generation of *n*_*b*_ linked batch codes of length ${\ell }_{b}+2$. Each individual batch code is to required to pass the set of filters specified above. The method we describe is suitable for the generation of a barcode library for use in a pooled experiment, i.e., when *n*_*b*_ ≪ *n*_*t*_. Later, we point out a trivial procedural modification that applies to the general setting when the user wishes to generate *N* barcodes without *n*_*b*_ ≪ *n*_*t*_. The Hamming filter requires that all pairs of generated batch codes satisfy a minimum Hamming distance of ${d}_{b}=\lfloor d\mathrm{/2}\rfloor$, if ${\ell }_{b}\le {\ell }_{t}$; otherwise the value of ${d}_{b}$ is $\lfloor (d-\mathrm{1)/2}\rfloor$. The generation process is iterative. The *i*-th batch code ($i\,=\,\mathrm{1,}\ldots ,{n}_{b}$) is generated by first generating a candidate batch code at random (i.e. according to our Markov model with $\delta \,=\,\mathrm{1/4}$ and $m\,=\,1$) and postfixing it with a 2 bp linker. The first base pair of the linker is chosen at random from among the three base pairs that do not occur in the last position of the batch code. The second base pair is chosen in the same manner except that it must not be in the first position of the linker. The purpose of the linker is to prevent homopolymers from forming between batch codes and target codes. The linked batch code is then checked to see whether it fails any of the filters. In the case of the minimum Hamming distance filter, the candidate must be checked against all perviously generated linked batch codes from $j\,=\,\mathrm{1,}\ldots ,i-1$. If the candidate passes all the filters, then it is welcomed as the *i*-th linked batch code; otherwise a new candidate is proposed and the same step repeated. This whole process is continued indefinitely until a set of *n*_*b*_ linked batch codes is generated.

### Target Code Generation

Let us now turn to the matter of generating *n*_*t*_ target codes of length ${\ell }_{t}$ given a pre-generated set of linked batch codes. The generation process begins with the selection of the optimal parameter for the Markov model of sequence generation using a grid search.

We employ a grid search on a set of *r* points ${\delta }_{i,r}=i/(r-\mathrm{1)}$ for $i=\mathrm{0,}\ldots ,r-1$ uniformly spaced over the unit interval to select an optimal value of *δ* in our Markov model. For each ${\delta }_{i,r}$, we generate a sample of *s* candidate target codes given the pre-generated batch codes. Each candidate target code is checked for whether it passes all filters when postfixed to each batch code. The proportion of successes averaged over *t* trials is recorded as the value of ${\delta }_{i,r}$. The grid point corresponding to the highest pass rate is taken as the optimum value of the model parameter *δ*.

From there the target codes are generated iteratively in much the same way as were the batch codes. One important difference, however, is that we use the Markov model to generate candidate sequences instead of at random. Note that if there is a linked batch code that when postfixed with the candidate target code fails to pass all of the specified filters, then the candidate is rejected; otherwise it is accepted as the *i*-th target code. As before, this process is continued until a set of *n*_*t*_ target codes are successfully generated.

### Data availability

The one billion barcode collection described in the paper is available for download at www.tupac.bio/publications.

## Algorithm Discussion

There are a number of points in regard to the workings of our method that merit discussion. First, the purpose of the linker is to prevent homopolymer continuation between the batch and target codes. The linker ensures that a homopolymer of maximum length 1 bp will be appended to any target code. Second, we show our optimal parameter selection scheme outperforms the simpler *δ* = 1/4 alternative in a number of simulated examples in Table 1. Third, the construction of barcode libraries through the separate generation of batch and target codes serves a useful algorithmic purpose. If the total number of barcodes in a library is given a *N*, without the use of our barcode structure the minimum number of Hamming comparisons required to generate a library works out to $N(N-\mathrm{1)/2}$, which is on the order of *N* ^2^. However, a library generated by our framework is constituted of $N={n}_{b}\times {n}_{t}$ barcodes in total. Therefore using our framework the minimum number of Hamming comparisons to generate the *n*_*t*_-th (final) target code is on the order of ${n}_{t}({n}_{b}-\mathrm{1)/2}+{n}_{t}({n}_{t}-\mathrm{1)/2}+{n}_{t}{n}_{b}={N}^{2}/{n}_{b}^{2}+N+{n}_{b}^{2}$, which is on the order of ${N}^{2}/{n}_{b}^{2}$, since ${n}_{t}=N/{n}_{b}$. This means that by using our framework the number of Hamming comparisons required, and therefore computational time, is greatly reduced compared with the conventional method. Note that the advantage is maximized when ${n}_{t}\approx {n}_{b}$, or in other words, when the number of batch codes is large.

**Table 1:** Runtimes for the generation of barcodes of length 25, 50, and 100 bp for different values of *m* using the Naïve framework, and two versions of our framework, Framework A and Framework B, as described in the Methods and Results sections. The library size in each case is *N* = 1,000,000. Framework A and Framework B were run with *n*_*b*_ = 100 batch codes and *n*_*t*_ = 10,000 target codes. Frameworks A and B outperform the Naïve framework in all cases. The runtimes are reported in [h]:mm:ss format. The runtimes for the Naïve framework are estimated as described in the main text.

Generation Framework	*m*	Length (bp)	Acceptance rate	Time
Naïve	2	25	0.00	649:00:00
Framework A	2	25	0.24	4:05
Framework B	2	25	0.36	2:24
Naïve	2	50	0.00	2146:00:00
Framework A	2	50	0.10	11:14
Framework B	2	50	0.49	1:54
Naïve	2	100	0.00	22295:00:00
Framework A	2	100	0.01	3:33:52
Framework B	2	100	0.39	2:41
Naïve	3	25	0.00	238:00:00
Framework A	3	25	0.49	2:10
Framework B	3	25	0.33	2:23
Naïve	3	50	0.00	322:00:00
Framework A	3	50	0.24	5:09
Framework B	3	50	0.46	2:01
Naïve	3	100	0.00	649:00:00
Framework A	3	100	0.18	7:12
Framework B	3	100	0.25	4:16
Naïve	4	25	0.00	191:00:00
Framework A	4	25	0.38	2:49
Framework B	4	25	0.45	1:56
Naïve	4	50	0.00	215:00:00
Framework A	4	50	0.34	3:54
Framework B	4	50	0.42	2:30
Naïve	4	100	0.00	259:00:00
Framework A	4	100	0.43	3:21
Framework B	4	100	0.50	1:58

### Computing Resources

All computations were run on a MacBook Pro laptop with a 2.6 GHz Intel Core i7 processor and OS 10.12.3.

## Results

In this section we show that our barcode generation framework (1) has a speed advantage over a naïve approach to barcode generation, (2) can generate a barcode library consisting of one million barcodes for use in a practical fragment antibody phage display screening experiment, and (3) can generate a general purpose barcode library consisting of one billion barcodes.

To determine the overall performance advantage of our framework, we compared a naïve barcode generation framework with two versions of our framework, Framework A and Framework B, as shown in Table 1. In the Naïve framework, nucleotide sequences are generated uniformly at random. Framework A employs our batch/target structure with the Markov chain parameter *δ* for nucleotide sequence generation fixed at 1/4. Framework B also uses our batch/target structure, but a grid search is used to find the optimum value of *δ* in the Markov chain model for the given constraints before the barcode library is generated, as described in the Methods section. Barcode libraries were generated under varying conditions using each framework in the context of a theoretical experiment to discover a new antibody drug candidate for macular degeneration by generating large numbers of variants of the existing drug Ranibizumab^19^. In each case a library of barcodes is generated for use in barcoded antibody fragment heavy chain genes that can be cloned in the pComb3x phagemid, expressed using phage display, and screened using a binding assay in combination with NGS. The experimental setup is as follows: we generated a number of libraries of size $N=\mathrm{1,000,000}$ with barcodes of length *L* = 25, *L* = 50, and *L* = 100. The *n*_*b*_ = 100 batch codes are of length ${\ell }_{b}=10$ and ${n}_{t}=10000$ target codes are of length ${\ell }_{t}=13$, ${\ell }_{t}=38$, and ${\ell }_{t}=88$, under the constraints that *d* = 4, and GC content from 35% to 65%. Sequences related to using the barcodes with the pComb3x phagemid, TCTAGA, TACCCGTACGACGTTCCGGACTACGCT, GAAGAC, AGGAGG, CACCATCACCATCACCAT, GAGCTC, and ACTAGT are blacklisted. We also attempted to generate an $N\mathrm{=1,000,000}$ barcode library with TagGD^17^ under the same constraints with *L* = 100 and *m* = 4, but the program was unable to generate the library after 24 hours of run time.

We compared Frameworks A and B for a number of values of *m*. Recall that *m* is the maximum allowable homopolymer length in a barcode. The total time required to generate a barcode library is mostly dependent on the pass rate of the barcodes through the filtering steps after a candidate barcode is generated. The results in Table 1 show that Framework B resulted in a significant speed up when compared with Framework A when the filtering constraint for maximum homopolymer length is short (i.e. *m* = 2) and the barcode length is long (i.e. *L* = 100 bp). This is to be expected as the Markov models were used specifically to prevent the generation of homopolymer sequences. However, when the maximum allowable homopolymer is long the Markov model and random nucleotide generator perform similarly, since a random nucleotide generator does not often produce very long homopolymers. The message here is that it is beneficial to resort to non-random nucleotide sequence generation when the goal is to generate sequences with a short homopolymer length. This is important because sequencing errors are prone to occur when long homopolymers are in play as we have described above. Barcode lengths of 25 bp were chosen from previous literature^8^, while 50 bp and 100 bp were chosen with a view towards future applications and to demonstrate the use cases for the Markov chain nucleotide generation model. The calculations for Naïve framework were stopped after 24 hours, as it was clear this method was not getting close to achieving the goal within a time being competitive our framework. Each simulation completion time was easily estimated by using linear regression to extrapolate completion time as a function of the number of barcodes generated on a double logarithmic scale.

As a further proof of concept, we generated a library consisting of one billion barcodes. This constitutes the largest library of barcodes yet reported to our knowledge. The library was generated with total barcode length of $50$ bp, with batch code of length $20$ bp, a $2$ bp linker, and a $38$ bp target code. Common restriction enzyme recognition site sequences for EcoRI, EcoRV, SalI, SmaI, XmaI, HindIII, BbsI, XhoI, and BgII were blacklisted so the library would be useful in multiple experimental settings. The generation time was approximately $10$ hours. The output is stored as a text file containing batch codes and a text file containing the target codes. We have provided a script that uses these two text files to output an *N* sized (up to one billion) library of barcodes to a new text file. These materials are freely available at www.tupac.bio/publications.

## Discussion

In this paper we report a framework to quickly generate large libraries of barcodes. Our framework follows in the tradition of previous approaches in so far as the number of barcodes, their length, the global minimum Hamming distance between barcode pairs, the maximum homopolymer length, and blacklisted sequences are subject to user customization. What distinguishes our framework from previous ones is that we are able to quickly generate much larger barcode libraries. This is possible because two distinct novelties underlie our methodology. The first is that we reduce the number of comparisons required for calculating the Hamming distances between a candidate code and the already accepted ones. The second is that we reduce the number of rejected candidate batch/target by generating them according to a Markov chain model that effectively caps the maximum homopolymer length in the sequence at a fixed value. This becomes more important as the barcode length becomes longer, since homopolymers are more probable to occur in long sequences. This framework has the added bonus of being particularly amenable to barcode design for pooled experiments.

There are a number of possible optimizations for speeding up the the calculation of Hamming distance that we leave as future work. The computational cost of calculating Hamming distance grows exponentially in the number of barcodes to be generated, making it the main bottleneck in the generation framework. There are a number of strategies that could be employed to mitigate this bottleneck in practice. First, storing DNA barcode sequences in binary form is one straightforward step to take. We stored sequences as strings in our implementation. Second, at a higher level the storage of sequences in a tree structure would cut down on the total number of individual comparisons required in computing Hamming distance. We stored sequences in a list data structure. Third, the previous two optimizations can be combined with parallelization for even faster generation. Lastly, if the size of the required library is small compared to the number of possible barcodes, it is also possible to estimate the Hamming distance between candidate barcodes and existing barcodes using sampling-based techniques.

Other general improvements in barcode library generation can be made through standardization of barcode design constraints for specific DNA sequencing and DNA synthesis technologies. This would allow for the generation of barcode libraries and generation methods that work reliably for a specific application. For example, when using Roche 454 sequencing the majority of errors are insertions and deletions^20^, when using Illumina the majority of errors are substitutions^21^, and other sequencing technologies can have significantly lower accuracy (92% in the case of the Oxford Nanopore MinION^22^). Therefore in practice selecting a barcode library or generating a new one that can satisfy the requirements of the specific application is nontrivial. In the case of the libraries we generated in the Results section, we envision these being used with the Illumina NextSeq. 500 or a comparable sequencer that uses SBS chemistry, which produces a majority of reads with an error rate of 0.1% or less^23^. The generated barcode libraries have a minimum Hamming distance of $m=4$, which means that there would need to be two errors in a given sequencing read to have difficulty matching the read to the correct barcode. Since the probability of two errors occurring in a read of $25$ bp or 100 bp is very low, there will be a very small number of erroneous reads that cannot be matched to the correct barcode, and these reads may be ignored during the analysis of sequencing data without significantly impacting the experiment.

Another important consideration when using barcodes designed in silico for *in vitro* experiments is the error rate introduced in manufacturing the physical DNA libraries. A high error rate in DNA synthesis could result in the manufacturing of erroneous barcodes that when used in an experiment, may result in identifying the incorrect barcode, or no barcode at all, during analysis of sequencing data. An example DNA manufacturer is Twist Bioscience, which has a synthetic DNA fragment error rate of $1$:3000 bp^24^. Therefore it is unlikely that more than four errors will be introduced in our barcodes when manufactured using this technology, and our selection of a minimum hamming distance of four in our simulations is robust enough to avoid barcode misidentification due to DNA synthesis errors. Furthermore we expect the DNA synthesis technology to improve, and the synthesis error rate can be expected to diminish along with the cost. As high-throughput screens using cheap synthetic DNA barcodes and data collection through sequencing becomes more common, we expect the methods for barcode generation, standardization, and analysis to grow in importance. Finally, a public database of barcode libraries with a standard method of cataloging the characteristics of each library would be of benefit for biologists selecting pre-generated barcode libraries.
